# Association of left ventricular longitudinal myocardial function with subclinical right ventricular dysfunction in type 2 diabetes mellitus

**DOI:** 10.1186/s12933-021-01404-5

**Published:** 2021-10-23

**Authors:** Saki Todo, Hidekazu Tanaka, Yuki Yamauchi, Shun Yokota, Yasuhide Mochizuki, Hiroaki Shiraki, Kentaro Yamashita, Ayu Shono, Makiko Suzuki, Keiko Sumimoto, Yusuke Tanaka, Yushi Hirota, Wataru Ogawa, Ken-ichi Hirata

**Affiliations:** 1 Division of Cardiovascular Medicine, Department of Internal Medicine, Kobe University Graduate School of Medicine, 7-5-2, Kusunoki-cho, Chuo-ku, Kobe, 650-0017 Japan; 2 Division of Diabetes and Endocrinology, Department of Internal Medicine, Kobe University Graduate School of Medicine, Kobe, Japan

**Keywords:** Type 2 diabetes mellitus, Right ventricular systolic function, Global longitudinal strain, Echocardiography

## Abstract

**Background:**

Left ventricular (LV) involvement in diabetic cardiomyopathy has been reported; however, only limited data exist on right ventricular (RV) involvement. Therefore, our purpose was to investigate RV systolic dysfunction and its association with LV longitudinal myocardial dysfunction in patients with type 2 diabetes mellitus (T2DM) and preserved LV ejection fraction (LVEF).

**Methods:**

We studied 177 T2DM patients with preserved LVEF and 79 age-, sex-, and LVEF-matched healthy volunteers. LV longitudinal myocardial function was assessed as global longitudinal strain (GLS), and RV systolic function was assessed as RV free-wall strain, and predefined cutoff values for subclinical dysfunction were set at GLS < 18% and RV free-wall strain < 20%, respectively.

**Results:**

RV free-wall strain in T2DM patients was significantly lower than that in normal controls (19.3% ± 4.8% vs. 24.4% ± 5.1%; P < 0.0001). RV free-wall strain in T2DM patients and LV longitudinal dysfunction was similar compared to that in T2DM patients without (19.0 ± 4.5% vs. 19.6 ± 5.0%, P = 0.40). Furthermore, multivariate logistic regression analyses showed that GLS was independently associated with RV systolic dysfunction as well as mitral inflow E and mitral e′ annular velocities ratio (odds ratio, 1.16; 95% confidence interval: 1.03–1.31; P < 0.05). Sequential logistic models evaluating the association of RV systolic dysfunction in T2DM patients showed an improvement in clinical variables (χ^2^ = 6.2) with the addition of conventional echocardiographic parameters (χ^2^ = 13.4, P < 0.001) and a further improvement with the addition of GLS (χ^2^ = 20.8, P < 0.001).

**Conclusion:**

RV subclinical systolic dysfunction was observed in T2DM patients with preserved LVEF and was associated with LV longitudinal myocardial dysfunction. Our findings may provide additional findings for the management of T2DM patients.

**Supplementary Information:**

The online version contains supplementary material available at 10.1186/s12933-021-01404-5.

## Background

Type 2 diabetes mellitus (T2DM) has come to be considered as an important contributor to the development of various types of heart failure (HF) [1, 2]. Left ventricular (LV) myocardial tissue abnormalities, such as myocardial fibrosis and myocyte hypertrophy, are observed even in patients with T2DM and preserved LV ejection fraction (LVEF). Diabetes-related cardiomyopathy, known as diabetic cardiomyopathy, possibly leads to HF with preserved ejection fraction (HFpEF). LV longitudinal myocardial dysfunction, as assessed in terms of lower global longitudinal strain (GLS), has been identified even in patients with T2DM and preserved LVEF but without overt coronary artery disease or HF [3–7] and should be considered the first marker of a preclinical form of diabetic cardiomyopathy. Furthermore, LV longitudinal myocardial dysfunction is strongly associated with poor outcomes in asymptomatic patients with T2DM and preserved LVEF [8]. Thus, the assessment of LV longitudinal myocardial function plays an important role in the better management of patients with T2DM and stage A HF.

The role of right ventricular (RV) systolic function has been increasingly recognized, and there is a growing body of evidence that RV systolic function is a powerful predictor of mortality in patients with various types of HF [9–12]. Several investigators have previously reported RV systolic dysfunction in T2DM patients with preserved LVEF [13–18]. However, the association between LV longitudinal myocardial function and RV systolic function in patients with T2DM and preserved LVEF remains uncertain. Therefore, the purpose of this study was to investigate the presence of subclinical RV systolic dysfunction in asymptomatic patients with T2DM and preserved LVEF without coronary artery disease and to investigate the association between LV longitudinal myocardial function and RV systolic function in such patients.

## Methods

### Study population

A total of 177 asymptomatic patients with T2DM and preserved LVEF (all ≥ 55%) without coronary artery disease who were admitted to Kobe University Hospital between June 2013 and March 2020 were retrospectively studied. The mean patient age was 61 ± 13 years, 83 patients (47%) were women, and the mean LVEF was 66% ± 5% (all ≥ 55%). All enrolled patients underwent an exercise stress screening test, such as a treadmill exercise or stress myocardial perfusion scintigraphy during hospitalization, and patients with an ischemic response were excluded. The preliminary exclusion criteria were as follows: (1) history of coronary artery disease; (2) previous history of open-heart surgery or congenital heart disease; (3) severe renal dysfunction defined as a glomerular filtration rate < 30 mL/min/1.73 m^2^; (4) uncontrolled hypertension with blood pressure > 180/100 mmHg; (5) more than moderate valvular heart disease; and (6) atrial fibrillation. The diagnosis of T2DM was based on World Health Organization criteria [19]. For comparison, a control group including 79 age-, sex-, and LVEF-matched normal subjects without T2DM or cardiovascular disease were randomly chosen from our database by the observers who were not involved in the echocardiographic analysis. This study was approved by the local ethics committee of our institution (No. B210127).

### Standard echocardiographic examination

All patients with T2DM and normal controls underwent transthoracic echocardiography. All echocardiographic data were obtained using a commercially available echocardiographic system (Vivid E9; GE Vingmed, Horten, Norway). Digital routine grayscale two-dimensional cine loops from three consecutive heartbeats were obtained at end-expiratory apnea from standard parasternal and apical views. The sector width was optimized to allow for complete myocardial visualization while maximizing the frame rate. Standard echocardiographic measurements were obtained in accordance with the current guidelines of the European Association of Cardiovascular Imaging [20].

### Speckle-tracking strain analysis

Two-dimensional speckle-tracking strain analysis was performed for each patient using dedicated software (EchoPAC version 113; General Electric Medical Systems, Milwaukee, WI, USA) to evaluate LV longitudinal myocardial function and RV systolic function. LV longitudinal myocardial function was assessed as GLS, and longitudinal speckle-tracking strain was calculated using an automated contouring detection algorithm, and manual adjustments of the region of interest were performed, if necessary. Longitudinal strain results for the individual clips were visualized in a color-coded format and combined in a bull’s eye plot. GLS was then determined as the average peak longitudinal strain of 18 LV segments and was expressed as an absolute value [20]. RV systolic function was assessed as RV free-wall strain, which was calculated by averaging each of the three regional peak systolic strains along the entire RV free-wall, and expressed as an absolute value [20]. In accordance with the current guidelines of the European Association of Cardiovascular Imaging, the predefined cutoff for LV longitudinal myocardial dysfunction and RV systolic dysfunction was set at a GLS < 18% and RV free-wall strain < 20%, respectively [20].

### Statistical analysis

Continuous variables are expressed as mean values with standard deviation for normally distributed data and median values with interquartile range for non-normally distributed data. Categorical variables are expressed as frequencies and percentages. The parameters of the two subgroups were compared using Student’s *t*-test or the Mann–Whitney *U* test, as appropriate. Proportional differences were evaluated using Fisher’s exact test. The initial univariate logistic regression analysis to identify univariate determinants of RV systolic dysfunction (RV free-wall strain < 20%) was followed by a multivariate logistic regression model using stepwise selection, with the P-values for entry into the model set at < 0.50. Sequential logistic models were performed to determine the incremental benefit of GLS in relation to RV systolic dysfunction using clinical variables, including age, sex, dyslipidemia, and estimated glomerular filtration rate (eGFR), and echocardiographic parameters, including LVEF, mitral inflow E and mitral e′ annular velocity ratio (E/e′), and left atrial volume index. A statistically significant increase in the global log-likelihood χ^2^ of the model was considered to represent an incremental predictive value. For all steps, a P-value < 0.05 was considered statistically significant. All analyses were performed using commercially available software (MedCalc, version 19.6; MedCalc Software, Mariakerke, Belgium).

## Results

### Baseline characteristics of patients with T2DM and controls

The baseline clinical and echocardiographic characteristics of the 177 patients with T2DM and 79 normal controls are summarized in Table 1. Clinical data showed that patients with T2DM were more likely to have a higher body mass index (25 ± 5 kg/m^2^ vs. 22 ± 4 kg/m^2^, P < 0.0001), higher systolic blood pressure (131 ± 20 mmHg vs. 125 ± 14 mmHg, P = 0.049), increased heart rate (70 ± 11 bpm vs. 66 ± 10 bpm, P = 0.013), and higher prevalence of hypertension (107 (60%) vs. 6 (8%), P < 0.0001) and dyslipidemia (105 (59%) vs. 6 (8%), P < 0.0001), while echocardiographic data showed that patients with T2DM were more likely to have a larger left atrial volume index (30.0 ± 8.4 mL/m^2^ vs. 27.1 ± 8.4 mL/m^2^, P = 0.02), LV mass index (81.7 ± 21.2 g/m^2^ vs. 71.5 ± 19.2 g/m^2^, P = 0.0004), and E/eʹ (11.0 ± 4.1 vs. 8.4 ± 2.5, P < 0.0001) and a smaller GLS (17.6 ± 3.1% vs. 20.5 ± 1.8%, P < 0.0001) and RV free-wall strain (19.3 ± 4.8% vs. 24.4 ± 5.1%, P < 0.0001) compared to normal controls.

**Table 1:** Baseline characteristics of T2DM patients and normal controls

Variables	T2DM patients (n = 177)	Normal controls (n = 79)	P value
Clinical characteristics			
Age, years	61 ± 13	58 ± 14	0.12
Gender (female), n (%)	83 (47)	45 (57)	0.14
DM duration, years	10 (2–16)	–	–
BMI, kg/m^2^	25 ± 5	22 ± 4	< 0.0001
Systolic blood pressure, mmHg	131 ± 20	125 ± 14	0.049
Heart rate, bpm	70 ± 11	66 ± 10	0.013
eGFR, mL/min/1.73 m^2^	74.0 ± 24.0	79.4 ± 26.2	0.13
HbA1c, %	8.8 ± 2.0	5.6 ± 0.5	< 0.0001
Comorbidities, n (%)			
Hypertension	107 (60)	6 (8)	< 0.0001
Dyslipidemia	105 (59)	6 (8)	< 0.0001
Diabetic-related comorbidities, n (%)			
Neuropathy	53 (30)		
Retinopathy	58 (33)		
Nephropathy	69 (39)		
Antidiabetic drugs, n (%)			
Insulin	80 (45)		
DPP-4I	89 (50)	–	–
GLP-1 RA	27 (15)	–	–
SU	38 (21)	–	–
α-GI	35 (20)	–	–
Thiazalidine	19 (11)	–	–
Metformin	87 (50)	–	–
SGLT2 inhibitors	20 (11)	–	–
Statins	72 (41)		
Calcium channel blockers	65 (37)		
β-blockers	25 (14)		
Echocardiographic Parameters			
LV end-diastolic volume, mL	69.3 ± 21.2	74.8 ± 22.5	0.06
LV end-systolic volume, mL	24.2 ± 9.7	26.5 ± 9.1	0.08
LVEF, %	66 ± 5	66 ± 5	0.54
LVMI, g/m^2^	81.7 ± 21.2	71.5 ± 19.2	0.0004
LAVI, mL/m^2^	30.0 ± 8.4	27.1 ± 8.4	0.02
E/A	0.8 ± 0.3	1.1 ± 0.3	< 0.0001
E/e′	11.0 ± 4.1	8.4 ± 2.5	< 0.0001
Tricuspid regurgitation velocity, m/s	2.1 ± 0.4	2.2 ± 0.3	0.009
GLS, %	17.6 ± 3.1	20.5 ± 1.8	< 0.0001
RV free-wall strain, %	19.3 ± 4.8	24.4 ± 5.1	< 0.0001

Values are mean ± SD for normally distributed data and median and interquartile range for non-normally distributed data, or n (%)

DM, diabetes mellitus; BMI, body mass index; eGFR, estimated glomerular filtration rate; DPP-4I, Dipeptidyl Peptidase-4 inhibitor; GLP-1 RA, glucagon-like peptide-1 receptors agonists; SU, Sulfonylureas; α-GI, α-glucosidase inhibitors; SGLT2, sodium glucose transporter type 2; LVEF, left ventricular ejection fraction; LVMI, left ventricular mass index; LAVI, left atrial volume index; e′, spectral pulsed-wave Doppler-derived early diastolic velocity from the septal mitral annulus; E, peak early diastolic mitral flow velocity; GLS, global longitudinal strain; RV, right ventricular

### Association between RV and LV GLS

**Fig. 1:**
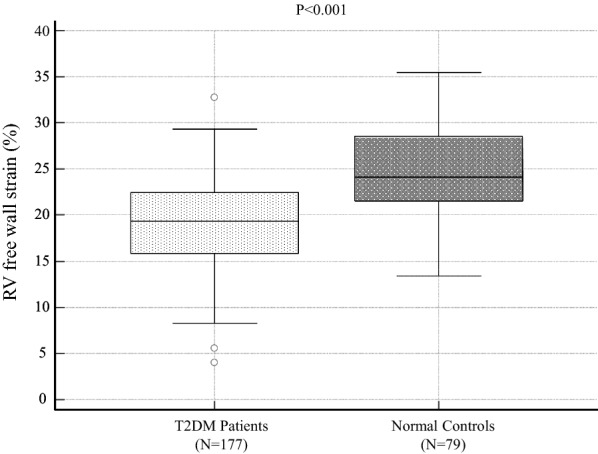
Comparison of RV free-wall strain between asymptomatic T2DM patients with preserved LVEF and normal controls, showing significant lower RV free-wall strain in asymptomatic T2DM patients with preserved LVEF

**Fig. 2:**
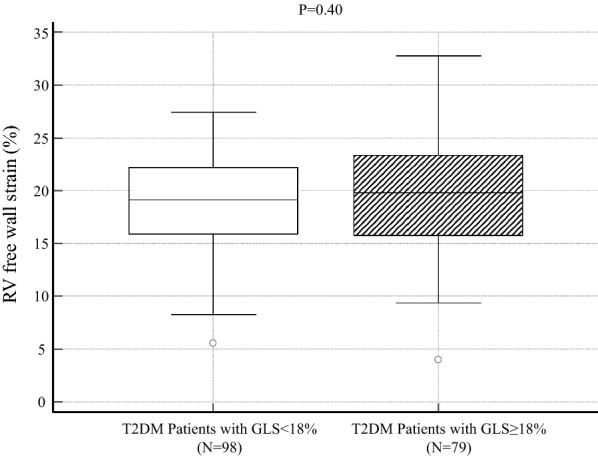
Comparison of RV free-wall strain in asymptomatic T2DM patients with preserved LVEF between those with GLS < 18% and GLS ≥ 18%, showing similar RV free-wall strain values

RV free-wall strain in patients with T2DM was significantly lower than that in normal controls as shown in Fig. 1 (19.3% ± 4.8% vs. 24.4% ± 5.1%; P < 0.0001). In addition, RV free-wall strain in patients with T2DM and LV longitudinal dysfunction (GLS < 18%) was similar compared to that in patients with T2DM without LV longitudinal dysfunction (GLS ≥ 18%), but this difference was not statistically significant (19.0 ± 4.5% vs. 19.6 ± 5.0%, P = 0.40; Fig. 2). Table 2 shows the results of the univariate and multivariate logistic regression analyses to identify RV systolic dysfunction in patients with T2DM. It is noteworthy that GLS was independently associated with RV systolic dysfunction as well as E/e′ (odds ratio: 1.16; 95% confidence interval: 1.03–1.31; P < 0.05). In addition, 54 T2DM patients were classified as having LV longitudinal dysfunction with RV systolic dysfunction, whereas, 44 T2DM patients were classified as having LV longitudinal dysfunction without RV systolic dysfunction. The characteristics of these two groups were almost similar except for body mass index, triglyceride, and high-density lipoprotein cholesterol. In T2DM patients with LV longitudinal and RV systolic dysfunction had significantly higher body mass index (28 ± 6 kg/m^2^ vs. 25 ± 6 kg/m^2^, P = 0.02) and triglyceride (180 ± 97 mg/dL vs. 142 ± 64 mg/dL, P = 0.03), and lower high-density lipoprotein cholesterol (48 ± 14 mg/dL vs. 55 ± 18 mg/dL, P = 0.04) compared to those with LV longitudinal dysfunction without RV systolic dysfunction.

**Table 2:** Univariate and multivariate logistic regression analyses to identify RV systolic dysfunction in T2DM patients

	Univariate			Multivariate		
	OR	95% CI	P value	OR	95% CI	P value
Age	1.02	0.99–1.04	0.18			
Female	1.21	0.67–2.18	0.53			
LDL cholesterol	1.00	1.00–1.01	0.27			
Systolic blood pressure	1.01	0.99–1.02	0.39			
eGFR	0.99	0.98–1.00	0.18			
GLS	1.08	0.98–1.19	0.13	1.16	1.03–1.31	< 0.05
LVEF	0.99	0.93–1.04	0.60			
E/e′	1.12	1.03–1.21	< 0.05	1.10	1.00–1.21	< 0.05
LAVI	1.04	1.01–1.08	< 0.05			

OR, odds ratio; CI, confidential interval; LDL-C, low-density lipoprotein cholesterol

All other abbreviations as in Table 1

The incremental benefits determined using sequential logistic models to identify the association between RV systolic dysfunction and clinical variables are shown in Additional file 1. One model based on clinical variables, including age, sex, dyslipidemia, and eGFR (χ^2^ = 6.2), showed an improvement with the addition of LVEF, E/eʹ, and left atrial volume index (χ^2^ = 13.4, P < 0.001) and a further improvement with the addition of GLS (χ^2^ = 20.8, P < 0.001).

**Fig. 3:**
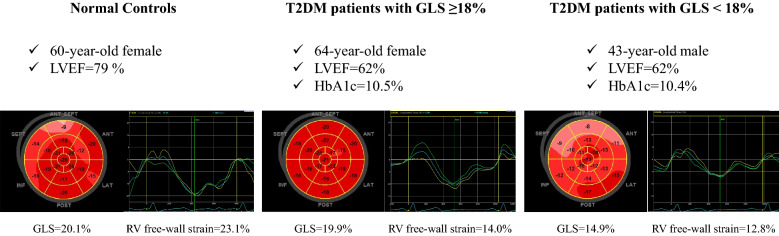
Representative cases of the polar plot longitudinal strain mapping and RV free-wall strain curves in a normal control, a T2DM patient with GLS ≥ 18% and a T2DM patient with GLS < 18%

Figure 3 shows representative cases of the polar plot longitudinal strain mapping and RV free-wall strain curves in a normal control, a T2DM patient with GLS ≥ 18%, and a T2DM patient with GLS < 18%.

## Discussion

The findings of our study indicate that subclinical RV systolic dysfunction was observed in asymptomatic patients with T2DM and preserved LVEF without coronary artery disease, and LV longitudinal myocardial function was also highly associated with RV systolic function in such patients.

### RV involvement by diabetic cardiomyopathy

LV systolic dysfunction due to left-sided HF is believed to be the most common cause of RV systolic dysfunction, which is independently associated with poor outcomes in various types of HF patients [9–12]. Currently, the relationship between LV and RV dysfunction has been considered as the following mechanism; (1) an increase in RV afterload through the development of pulmonary arterial hypertension secondary to chronic pulmonary venous hypertension; (2) possibility of bi-ventricular cardiomyopathic process such as simultaneous involvement of both RV and LV myocardium. Similar to patients with HFrEF, RV systolic dysfunction defined by RV fractional area change < 35% was common in patients with HFpEF and was associated with poor outcomes [21]; and (3) Ventricular interdependence via LV septum. RV systolic function was associated with septal dysfunction and limited pericardial flexibility, neurohormonal interactions, and reduced RV coronary perfusion secondary to decreased systolic driving pressure [22, 23].

T2DM is considered an independent predictor of mortality and contributes to the development of HF, even in patients with preserved LVEF in the absence of significant coronary artery disease and hypertension [24, 25]. Thus, the detection of subclinical LV dysfunction has become increasingly important in the management of asymptomatic patients with T2DM and preserved LVEF. This phenomenon is known as diabetic cardiomyopathy and has a complex and multifactorial pathogenesis. LV involvement in diabetic cardiomyopathy is well known; however, only limited data exist on RV involvement in diabetic cardiomyopathy in patients with T2DM and preserved LVEF. Several investigators have also previously reported RV systolic dysfunction in T2DM patients with preserved LVEF [13–18]. However, the association between LV longitudinal myocardial function and RV systolic function in patients with T2DM and preserved LVEF remains uncertain. As has just been described, the interrelationship of biventricular systolic dysfunction in patients with HF has been well discussed; however, the interrelationship of biventricular subclinical systolic dysfunction in patients with preserved LVEF, such as stage A HF, including T2DM, has not been completely elucidated. All proposed mechanisms leading to LV involvement in diabetic cardiomyopathy are systemic changes and therefore might hamper RV function. RV involvement in diabetic cardiomyopathy might be important because the right ventricle has a substantial contribution to overall myocardial contractility. In addition, the prevalence of cardiac conduction abnormalities is increased in patients with diabetes [26], and RV dysfunction and fibrosis are associated with lethal ventricular arrhythmias, sudden death, exercise limitation, and impaired RV cardiac output [27]. Tadic et al. showed that RV free-wall strain in patients with T2DM was significantly lower than that in age- and sex-matched healthy controls despite normal LVEF [28]. In this study, RV free-wall strain in asymptomatic patients with T2DM and preserved LVEF was significantly lower than that in age-, sex-, and LVEF-matched normal controls, and GLS was independently associated with RV free-wall strain in asymptomatic patients with T2DM and preserved LVEF. Taken together, subclinical RV and LV systolic dysfunction may also be impaired simultaneously, but our data suggest that in asymptomatic patients with T2DM and preserved LVEF without coronary artery disease, subclinical RV systolic dysfunction may primarily be a consequence of subclinical LV systolic dysfunction.

### Clinical implication

HF is a global public health problem, and the number of hospitalized patients due to HF is increasing, which is one of the most important issues for the management of HF [29], in what has been called the “HF pandemic.” Although the identification of individuals with stage A HF is potentially useful for the implementation of HF prevention strategies, not all patients with stage A HF develop LV structural heart disease or symptomatic HF, which can lead to advanced HF stages. LV longitudinal myocardial dysfunction, as assessed in terms of low GLS, can first appear in stage A HF, which suggests the importance of GLS assessment for detecting subclinical LV dysfunction in this subclinical stage. Thus, GLS-guided management for stage A HF may result in not only the improvement of individual comorbid diseases, but also the prevention of future development of LV structural heart disease and symptomatic HF. In addition to LV longitudinal myocardial dysfunction in asymptomatic patients with T2DM and preserved LVEF, RV systolic dysfunction was present and was independently associated with LV longitudinal myocardial dysfunction. Thus, considering the interrelationship of biventricular subclinical dysfunction in asymptomatic patients with T2DM and preserved LVEF could contribute to a better management of patients with stage A HF, including T2DM. Our findings suggest that established cardioprotective drugs, including novel cardioprotective medications such as sacubitril/valsartan and sodium-glucose cotransporter 2 inhibitors, may have potential as a new therapeutic strategy for asymptomatic patients with T2DM, preserved LVEF, and biventricular subclinical dysfunction.

## Study limitations

This was a single-center retrospective study, and was a cross-sectional study design so that there was only small evidence of the relationship between T2DM and RV dysfunction. Thus, prospective multicenter studies with larger patient populations and longitudinal data are needed to further assess our findings. Furthermore, the assessment of GLS and RV free-wall strain after treatment of T2DM was not part of this study because only a small number of patients were available for follow-up.

## Conclusion

RV subclinical systolic dysfunction was observed in asymptomatic patients with T2DM and preserved LVEF without coronary artery disease and was associated with LV longitudinal myocardial dysfunction. Our findings may provide additional findings for the management of T2DM patients.

## Supplementary Information

**Additional file 1. **The incremental benefits determined using sequential logistic models to identify the association of RV systolic dysfunction, showing that one model, based on clinical variables including age, gender, dyslipidemia, and eGFR, showed an improvement with the addition of LVEF, E/e′ and left atrial volume index and a further improvement with the addition of GLS.

## Data Availability

Data sharing not applicable to this article as no datasets were generated or analyzed during the current study.

## Abbreviations

E/e′: Mitral inflow E and mitral e′ annular velocities ratio
eGFR: Estimated glomerular filtration rate
GLS: Global longitudinal strain
HF: Heart failure
HFpEF: Heart failure with preserved ejection fraction
LV: Left ventricular
LVEF: Left ventricular ejection fraction
RV: Right ventricular
T2DM: Type 2 diabetes mellitus
