# Supplementary figures and images for: Association of left ventricular longitudinal myocardial function with subclinical right ventricular dysfunction in type 2 diabetes mellitus
Source: Cardiovasc Diabetol. 2021 Oct 23;20:212. doi: 10.1186/s12933-021-01404-5 (PMC8542339; doi:10.1186/s12933-021-01404-5)

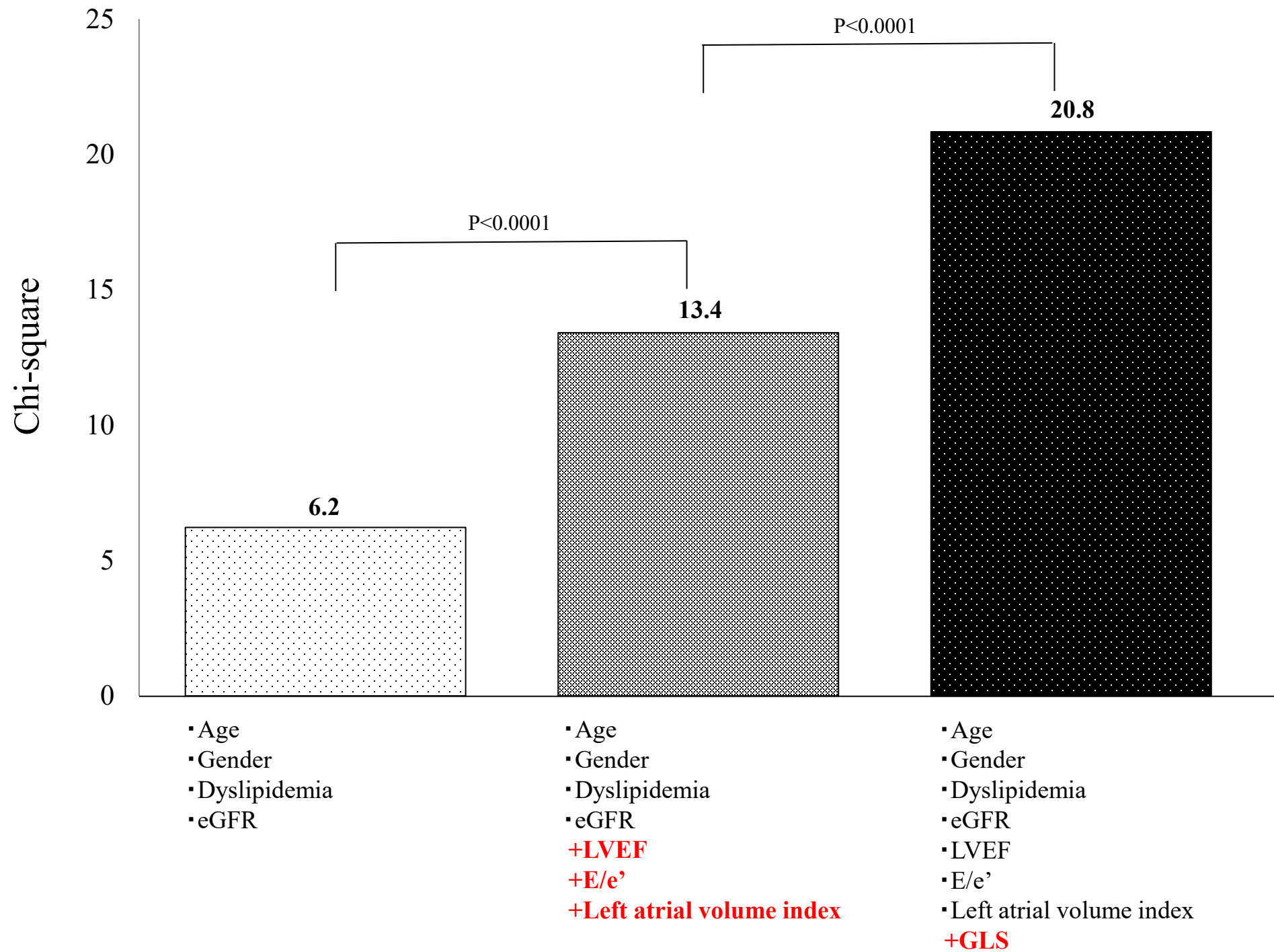

Supplement: Supplementary file 1 — Additional file 1. The incremental benefits determined using sequential logistic models to identify the association of RV systolic dysfunction, showing that one model, based on clinical variables including age, gender, dyslipidemia, and eGFR, showed an improvement with the addition of LVEF, E/e′ and left atrial volume index and a further improvement with the addition of GLS. [file 12933_2021_1404_MOESM1_ESM.pdf]
